# Effects of prevalence and feedback in the identification of blast cells in peripheral blood: expert and novice observers

**DOI:** 10.1186/s41235-025-00632-7

**Published:** 2025-06-15

**Authors:** Wanyi Lyu, Jennifer S. Trueblood, Jeremy M. Wolfe

**Affiliations:** 1 Department of Biology, Centre for Vision Research, York University, 4700 Keele Street, Toronto, ON M3J 1P3 Canada; 2 Department of Psychological and Brain Sciences and Cognitive Science Program, Indiana University, Bloomington, 47405 USA; 3 Visual Attention Lab, Brigham and Women’s Hospital, Boston, MA 02135 USA; 4 Departments of Ophthalmology and Radiology, Harvard Medical School, Boston, 02115 USA

**Keywords:** Perceptual decision making, Low prevalence effects, Prevalence-induced concept change, Expertise, Feedback

## Abstract

Low target prevalence affects perceptual decisions on both simple and complex stimuli. Without prior knowledge of how often targets may appear, trial-by-trial accuracy feedback modulates the effects of low prevalence partially by providing observers with information about the target base rate. Using simple colored dots, Lyu (PBR 28:1906–1914, 2021) found that at low prevalence, observers demonstrate a classical low prevalence effect (LPE) when receiving feedback. This involves a conservative shift of the decision criterion where observers are less likely to call an ambiguous item a target. In the absence of feedback, observers adopted more liberal criteria and became more likely to classify an item as a target, producing a Prevalence-Induced Concept Change (PICC, Levari et al., Science 360:1465–1467, 2018). The present study examines whether the effects of low prevalence and feedback are modulated by expertise. Novice (*n* = 26) and expert (*n* = 24) observers performed a cancer cell discrimination task. The prevalence of cancerous “blast cells” and the presence or absence of trial-by-trial accuracy feedback were manipulated. Unsurprisingly, medical professionals performed better than trained novices. Importantly, both experts and novices showed an LPE with feedback, although that LPE was weaker in experts, suggesting expertise may modulate the size of the LPE. Low prevalence had little effect on the criterion in the absence of feedback in this setting. For both novices and experts, initial exposure to trials with feedback influenced criteria in subsequent no feedback conditions. Interestingly, experts showed a conservative criterion at the start of the experiment, even without having experienced a feedback block. This could reflect previous training or working in a low prevalence setting. Our study shows the interactions of the effects of low prevalence, feedback, and expertise on perceptual decisions and provides direct evidence for prevalence and feedback effects on expert decisions.

**Supplementary Information:**

The online version contains supplementary material available at 10.1186/s41235-025-00632-7.

## Significance statement

Real-world identification of ambiguous stimuli is influenced by several factors, including target “prevalence”. How likely is it that X is present in this sort of stimulus? For example, how likely is it that one of your neighbors is a professional athlete? How likely is it that a piece of carry-on luggage contains a gun? It has been shown that observers are conservative in making positive identifications of rare items, leading to an increase in misses (The low prevalence effect—LPE). Reliable trial-by-trial accuracy feedback modulates the LPE. In the absence of feedback or prior knowledge of the prevalence rate, the LPE can be smaller or even reversed. Do these effects occur when experts assess items in their domain of expertise? We compared medical professionals and novices in categorizing blood cells as normal or abnormal “blast cells”. Observers had no prior knowledge of the prevalence rate in our experiment. Both groups showed the LPE, though it was smaller in the expert group. Both groups showed little or no LPE in the absence of feedback. Thus, expertise does not change the direction of the effects of low prevalence and feedback.

## Introduction

We spend a great amount of time evaluating potentially ambiguous stimuli: Is that black blob a bear? Is that my coffee cup? Is this spot on this patient’s CT scan a cancerous lesion? The base rate or ‘prevalence’ of these targets differs widely. Some are quite rare (I have never seen a bear around here.). Others are quite common (The coffee cup is on my desk. Such a cup is usually mine.). Target prevalence is known to influence search and decision processes (Colquhoun & Baddeley, 1967; Horowitz, 2017; Wolfe et al., 2005). Typically, low prevalence is associated with conservative decision criteria (few positive responses—correct or incorrect), while high prevalence is associated with more liberal criteria (fewer negative responses; Wolfe & Van Wert, 2010). Recently, Levari et al. (2018) described the opposite result: a shift to a more liberal criterion at low prevalence. Thus, low target prevalence can bias our performance reliably in two opposite directions. In the absence of prior knowledge about the target prevalence, the direction of the effect of low prevalence can be modulated by the availability of trial-by-trial feedback on the response accuracy (Lyu et al., 2021). The present paper examines whether the two opposite effects of low prevalence can be reliably reproduced in a medical image perception task in which observers are asked to discriminate cancerous cells from normal cells, and whether novices and experts show similar responses to prevalence and feedback when both groups view stimuli from the experts' domain of expertise.

The effects of low target prevalence and feedback can be demonstrated in a simple experiment. In the version introduced by Levari et al (2018), observers are shown a single-colored dot from a blue-purple continuum and instructed to judge whether that dot is blue or not. The prevalence of dots from the blue end of the continuum is varied. In the standard version of the experiment, initial prevalence is 50% and declines to a lower level over the course of the experiment. In the absence of feedback, when blue dots become rare, observers expand their definition of 'blue' and categorize more of the ambiguous dots as blue. This effect is called the Prevalence-Induced Concept Change (PICC; Levari et al., 2018) and represents a ‘liberal’ shift of the decision criterion in signal detection theory. PICC occurs in various contexts: when threatening faces become rare, observers categorize more neutral faces as threatening; when unethical scientific proposals become rare, observers judge more ethically ambiguous proposals as unethical (Levari et al., 2018). Conversely, in the presence of feedback, when blue dots become rare, observers adopt a more conservative criterion and categorize fewer dots as blue, consistent with the classic low prevalence effect (LPE). In visual search tasks, the LPE typically appears as an elevated false negative/miss error rate at low prevalence (Wolfe et al., 2007). When feedback was provided, effects of low prevalence have been consistently found in laboratory tasks where observers search for a T among Ls (Rich et al., 2008), and in the real-world when radiologists identify cancerous lesion (Evans et al., 2011, 2013), Traffic Security Administration workers look for a threat in carry-on luggage (Wolfe et al., 2013), and in tasks where individuals match fingerprints and faces (Bindemann et al., 2010; Davis et al., 2021; Papesh et al., 2018; Weatherford et al., 2020).

Both PICC and LPE can be seen as forms of a base rate problem in which the performance is influenced by the base rate or prevalence of the target, even though that base rate does not alter the visibility or physical properties of the stimulus (Bar-Hillel, 1980; Lyu et al., 2021; Trueblood et al., 2021). Feedback indirectly informs observers about the target base rate. If you say “no” most of the time and you are given feedback that you are correct most of the time, targets must be quite rare. When feedback is absent, observers can only infer the base rate from their own response rate. If you say “no” most of the time, you can infer either that targets are rare or that you are saying “no” too often. The observers’ level of confidence in their responses will make a difference. If you are sure about your evaluation of scientific proposals, for example, you don’t need external feedback to assess the prevalence of unethical proposals.

It is worth pointing out that among the decision tasks where observers exhibit LPE or PICC effects, some tasks have clearly specified reference standards, whereas others do not. For example, it is fairly unambiguous to most observers whether a letter is a T or an L or whether an item in luggage is a gun. However, when deciding whether a color is blue or whether a scientific proposal is unethical, the reference standard becomes less clear and can vary among individuals. Real-world tasks such as detecting cancer on CTs and classifying cells (reported in this paper) lie somewhere in the middle of the continuum. The reference standard for these visual tasks is less clearly defined due to the complexity of the visual signal and the complexity of the biological systems. While no study has systematically examined the effects of low prevalence in stimulus with or without a clear reference standard, LPE appears to be more robust and consistent among different stimulus types than the PICC effect.

In the presence of trial-by-trial feedback, low prevalence consistently produces LPE for stimuli with a clear reference standard (e.g. T vs L search (Rich et al., 2008), search for guns in bags (Wolfe & Wert, 2010; Wolfe et al., 2013)), for those without (search for blue color among purple and sharp shape from round shape (Lyu et al., 2021)), and for complex real-world stimuli (Face and fingerprint (Growns & Kukucka, 2021; Papesh et al., 2018; Weatherford et al., 2020)). When trial-by-trial feedback is absent, LPE still occurs if the target base rate is known. Evans et al. (2011, 2013) asked experts to categorize medical images and reported LPE in the absence of feedback. Experts either selected low prevalence values that match those in the clinical context or directly inserted positive cases into the clinical workflow. In those cases, observers’ knowledge of the standard target base rate probably influenced decisions. Two additional studies examined experts’ decision-making by directly manipulating their expectations of target prevalence. Trueblood et al. (2021) reported LPE when observers (novices and experts) were informed of the prevalence rate at the start of each block of trials, showing that explicit knowledge of the base rate can produce an LPE when prevalence is low. Littlefair et al. (2016) provided different prevalence information (low to high) to three groups of radiologists while they viewed the same set of chest radiographs. The study reported no significant changes in the radiologists’ sensitivity and a reduction in specificity (the ability to correctly identify negative cases) (Littlefair et al., 2016). Together, these studies show that, in the absence of feedback, prior knowledge of low prevalence can also affect the decision and produce an LPE.

If both feedback and prior knowledge on base rate are absent and if the stimuli do not have a clear reference standard, the PICC effect is robust with testing using visual (e.g., color categorization on a continuum, Levari et al., 2018; Lyu et al., 2021) and audio stimuli (Woodard et al., 2021). For stimuli with a somewhat clearer reference standard, less consistent results have been reported. When participants judged whether photographs of two faces were from the same person, without trial-by-trial feedback, Davis et al. (2021) found a liberal criterion shift when observers were not informed of the base rate, Papesh et al. (2018) found a small PICC effect (lower miss rate and higher false alarm rate), whereas Weatherford et al. (2020) found no significant change in decision criterion. Overall, in low prevalence detection or visual search tasks, LPE has been found consistently, while PICC appears to be more inconsistent and weaker in comparison.

The goal of the present study is to examine whether the effects of low prevalence and feedback are modulated by expertise using a cancer cell identification task. Robust prevalence effects have been demonstrated by several previous studies showing that experts are not exempt from the effects of low prevalence even when viewing the stimuli in their domain of expertise (Evans et al., 2013; Wolfe et al., 2013). However, few studies directly examined the interaction between expertise and feedback in low prevalence decisions. Experts and novices might respond to feedback differently. The extensive experience and knowledge might deter experts from shifting decision criteria when encountering negative feedback. Alternatively, experts might adjust their decision-making process differently from novices at low prevalence. The LPE has been modeled using the framework of the diffusion decision model (Ratcliff, 1978; Ratcliff et al., 2016). In that context, it has been shown that, while novices have a stronger tendency to shift the starting point of evidence accumulation (e.g., adopt a tendency to select one decision over the other), experts are more prone to change the mean rate of evidence accumulation. This suggests that experts may be evaluating information more carefully in extreme prevalence situations (Trueblood et al., 2021).

Prevalence effects on the cancer cell identification task used here were previously studied by Trueblood et al. (2021). They gave observers explicit base rate information, but not trial-by-trial feedback. In the present study, with a new population of experts, we directly manipulate both prevalence and feedback. The results show that feedback modulates low prevalence effects in both novice and expert observers. The study serves to bridge findings in basic research and their impacts in real-world tasks.

## Experiment 1: blast cell detection with novice observers

### Methods

#### Preregistration

Experiment 1 was preregistered on the Open Science Framework (https://osf.io/k2ngv/). The raw data files are also available at this location.

#### Observers & power

Prior experiments (Lyu et al., 2021) have an effect size that can be conservatively estimated to be ~ 0.8 for differences between high and low prevalence blocks. Some effects in the experiments proposed here may have more subtle effects, so we calculate power based on an effect size of 0.6. Detecting a difference of this magnitude between the high and low prevalence blocks, as measured by a two-tailed t-test, requires 24 observers to achieve an alpha of 0.05 and a power of 0.8. We planned to test observers until we had 24 observers after exclusions. We planned to exclude observers who did not pass the training section (average accuracy below 80%) and to replace any observers whose data indicated that they did not do the task but merely guessed or used one response (all ‘blast’ for example). In fact, no observers needed to be excluded based on these criteria. As a result, we collected data from 26 observers (23 female, 3 male) with an average age of 27 ± 9 years. Detailed demographical information can be found on the experiment OSF page.

Novices with no prior knowledge in hematopathology participated in Experiment 1. The observers were recruited from the Brigham & Women’s Hospital Visual Attention Lab volunteer pool. All observers had 20/25 vision or better with correction and passed the Ishihara Color Test (Ishihara, 1990). All observers gave informed consent prior to participating in the experiment and were paid $12/hour. Procedures were approved by the Institutional Review Board at Brigham and Women’s Hospital (IRB #2007P000646). All observers were naïve to the purpose of the experiment.

#### Stimuli & procedure

**Fig. 1:**
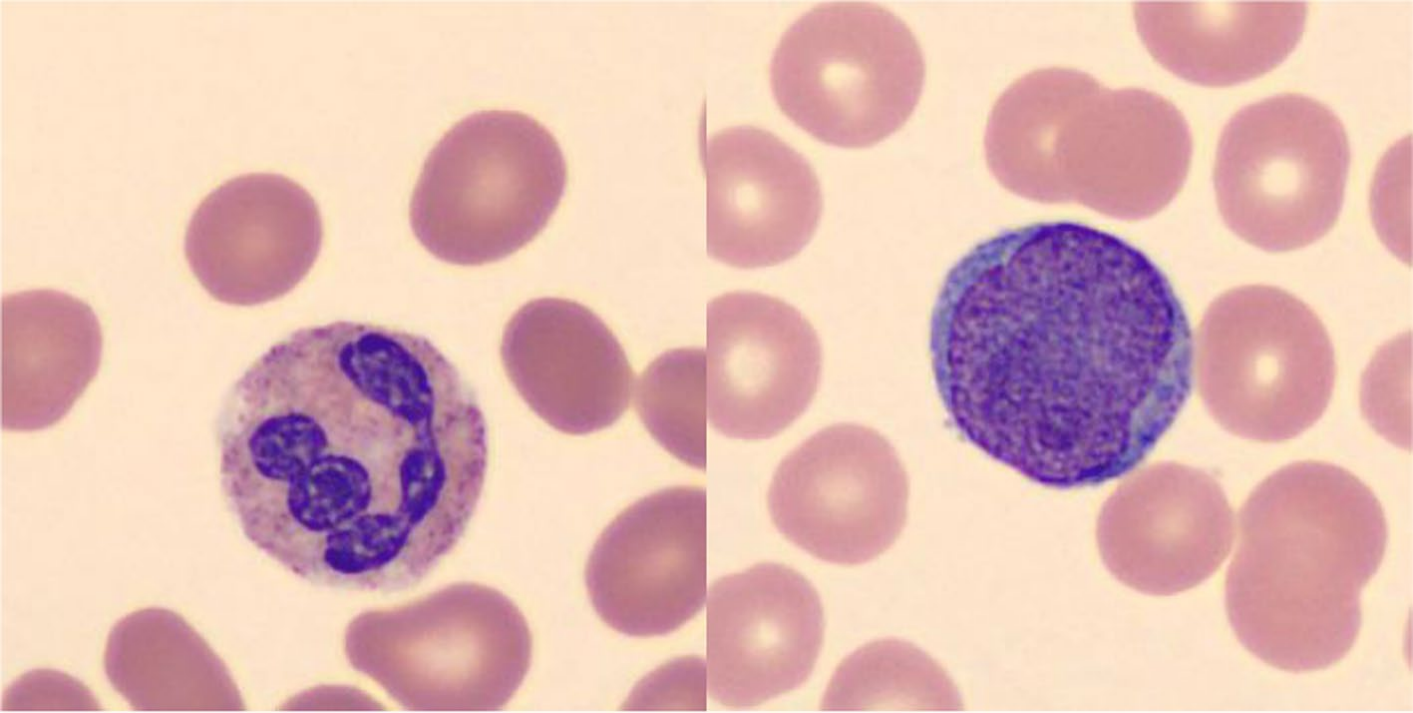
Examples of white blood cell stimuli used in the study. The white blood cell is the largest cell in the image, positioned near the center with a purplish core. The smaller cells in the background are red blood cells. (Left: non-blast cell; Right: blast cell)

The stimuli were 748 digital images of Wright-stained white blood cells (Fig. 1) taken from anonymized patient peripheral blood smears at Vanderbilt University Medical Center (VUMC). They were provided by J.T. and used in a previous study (Trueblood et al., 2018). The images were taken by an automated digital cell morphology instrument (CellaVision DM96, CellaVision AB, Lund, Sweden) and were evaluated by three hematopathology experts from the faculty of the Department of Pathology, Microbiology and Immunology at VUMC. The raters first identified each image as a blast or non-blast cell and then provided a difficulty rating for the image on a 1–5 scale (see Trueblood et al. (2018) for details of the rating procedure). We had a three-way agreement on 547 images. We used all 748 images in experiment 1 and 2 due to the limited number of stimuli available.

To sort the images along a continuum that goes from non-blast to blast, we first transformed the original response and 5pt difficulty rating from the hematopathology faculty into a 10pt scale that goes from least blast (1) to most blast (10). On the transformed scale, 1–5 are non-blast cells and 6–10 are blast cells. In this way, the cells with ratings at the two ends of the continuum are easily classified cells, whereas those with ratings in the middle of the continuum are difficult, ambiguous-looking cells. We then re-binned the average transformed ratings from the three raters into 6 groups (1-least blast, 6-most blast). Because some categories have more images than others, we used 6 groups so that there would be at least 50 images in each group. Twenty images from each group were randomly selected every 120 trials with replacement and used in the main experiment. Because some groups have more images than others, the likelihood that an image will appear more than once in the experiment differs for each category (Table 1). We examined the results when the analysis was limited to only repeated trials and found no qualitative changes in the pattern of results (See Supplementary Materials). Thus, we will not include repeated vs unrepeated as a factor in the analysis.

**Table 1:** The number of unique stimuli in each category and the percentage of images that appeared more than once Experiment 1 & 2

Category	Number of images	% Repeated images in Exp. 1 with novices	% Repeated images in Exp. 2 with experts
1	177	57.2%	58.0%
2	107	77.6%	78.2%
3	88	84.8%	85.9%
4	50	73.8%	76.1%
5	84	53.0%	52.7%
6	242	22.8%	22.6%

The experiment was written in JavaScript and hosted online. Participants could access the experiment using any web browser, using the URL we provided. The observers were screened virtually and monitored during the study by an experimenter over Zoom. The online nature of the experiment means that we did not have control over the viewing conditions or the screen type, but we have no reason to believe that these factors would differ systematically between conditions.

Before the experiment, observers saw instructions explaining that they would be shown images of white blood cells, and their task was to decide whether or not each image depicted a blast cell. They were told that each image showed either a blast or a non-blast cell, and that a blast cell is a pathological white blood cell whose presence is often a sign of blood cancers such as leukemia. No verbal instruction on the characteristics of blast cells was given to avoid limiting observers’ attention to only the mentioned features. No information on the prevalence of the blast cells was provided. Observers viewed 12 labeled exemplar images (6 blast and 6 non-blast images drawn randomly) and completed two training blocks, easier followed by harder, before the main experiment. On each training trial, observers saw one image at a time for as long as they needed and judged whether the image showed a blast cell by selecting the corresponding button on the screen. They received trial-by-trial accuracy feedback on their response. Only categories 1 & 6 were used in the Easy training block. The images were drawn from the blast and non-blast image pools with equal probability. To pass the Easy training block, observers had to complete at least 20 trials and receive a rolling accuracy of 80% (i.e., observers kept going until they got 16 correct out of the last 20 trials). The Hard block was identical to the Easy block, except that the harder to classify images from categories 2 & 5 were used. Again, observers completed at least 20 trials and achieved a rolling accuracy of 80% to pass. We excluded images from categories 3 & 4 from the training blocks to prevent exposing and/or overtraining observers with the hardest images. We expected that any effect of prevalence would be most pronounced for these ambiguous images and reserved them, entirely unseen, for the testing block.

The main experiment had four within-participant conditions: (feedback and no-feedback) X (higher prevalence (50%) and lower prevalence (20%)). Observers were tested on a block of 360 trials with feedback and another 360 without feedback, with no rest in between. The order in which observers experienced feedback and no feedback conditions was randomized. Within a block of 360 trials, the first 120 trials were high prevalence (50%). Images were chosen randomly from the blast and non-blast cell categories with the possibility of replacement. Trials 121–360 were low prevalence trials where 20% of the images were drawn from the blast categories and 80% from the non-blast categories.

On each trial, one cell image was presented at the center of the screen for 2000 ms. The stimulus had a width of 300 pixels, equivalent to 7.6 degrees of visual angle at a viewing distance of approximately 60 cm on a typical computer screen. The image appeared on a solid white background. Observers were instructed to press one key if they judged the image to depict a blast cell and another key if they judged it to depict a non-blast cell. They received trial-by-trial accuracy feedback in the feedback condition and were simply informed that their response had been recorded in the no-feedback condition. There was no resting time between trials in a block. Novice observers took on average an hour to finish the experiment.

#### Data analysis

As in prior work on this topic, we used signal detection measures of criterion and d’ to evaluate the effect of target prevalence and feedback. D’ is defined as:

z(True positive rate)–z(False Positive rate).

where z is the inverse normal transformation of the rate. (True positive = “hit”; False positive = “false alarm”). The criterion is defined as:

(z(True positive rate) + z(False Positive rate))/−2.

D’ is an index of the ability to discriminate target from non-target stimuli where 0 is chance responding. Criterion is an index of the bias in responding with values greater than zero indicating a bias to say non-target more often (“conservative”), while a negative criterion indicates a bias to say target (“liberal”).

### Results

Twenty-six observers participated in the experiment. One observer was excluded due to incomplete data. The remaining 25 observers had an above-chance average accuracy of 67.7% (*t*(24) = 56.27, *p* < 0.0001). Accuracies in feedback (68.6%) and no feedback (66.8%) conditions were not reliably different (*t*(24) = 1.79, *p* = 0.086). We removed trials with reaction times shorter than 200 ms and longer than 4000 ms (1.2% of all trials) from further analysis. For responses less than 200 ms, it is unlikely that observers could fully process the image and were likely guessing. For responses longer than 4000 ms, it is unclear whether observers were paying attention during those trials.

**Fig. 2:**
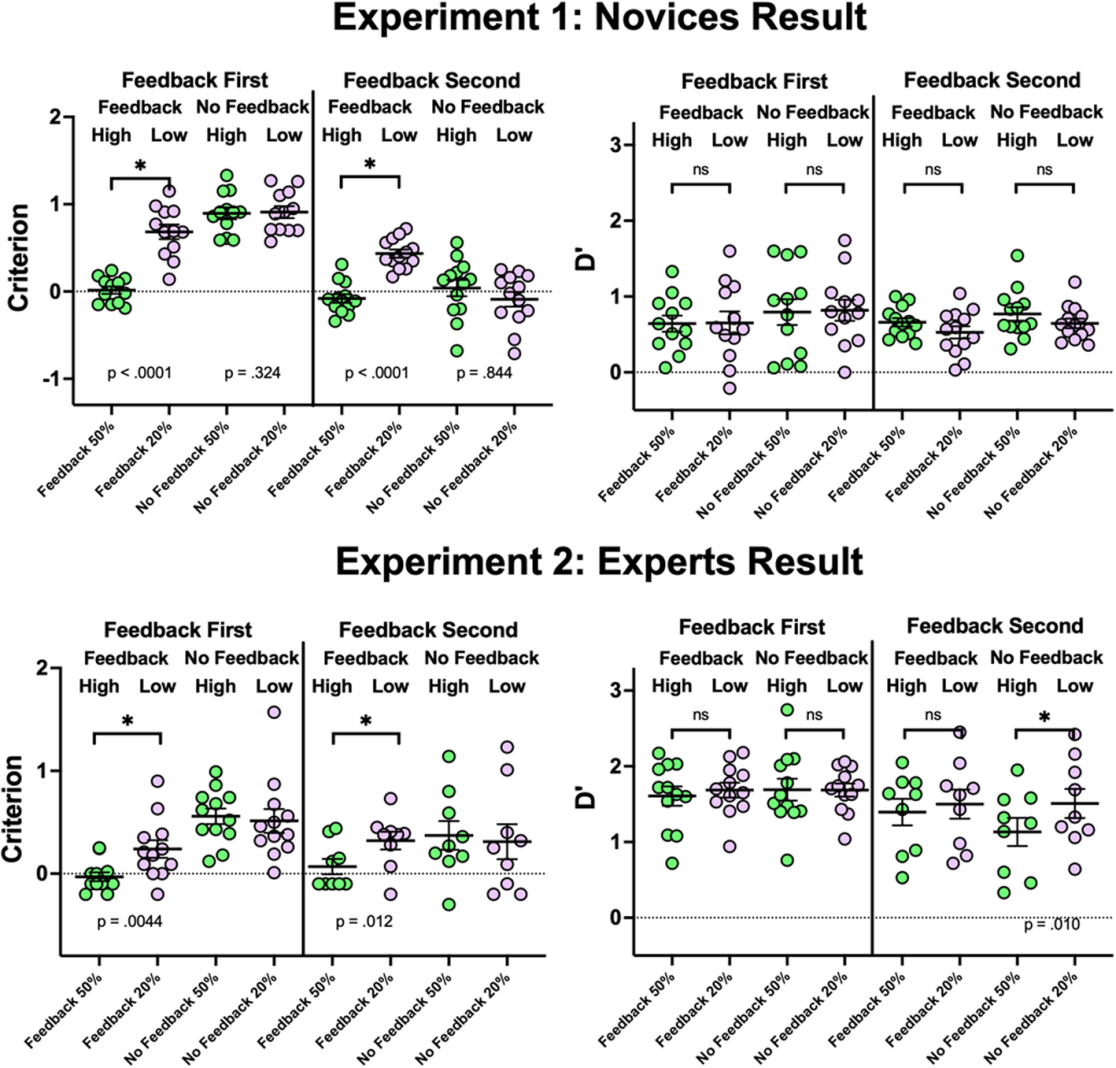
Criterion (left column) and d’ values (right column) for each observer in Experiment 1 (upper panel) & 2 (lower panel). High and low prevalence, feedback and no-feedback conditions are plotted separately for observers who received the feedback condition first and second. *P*-values show results for paired t-tests, testing against the null hypothesis that there is no effect of prevalence on criterion or d’. Error bars show ± 1 SEM

Results are shown in the upper row of graphs in Fig. 2. Observers are separated into two groups based on the order in which they received the feedback conditions. Previous work (Lyu et al., 2021) has shown that receiving the feedback condition first can shift the criterion in the subsequent no feedback condition. In the present experiment, when feedback comes first (*n* = 12), a two-way ANOVA with prevalence and feedback as factors shows significant effects of both prevalence (*F*(1, 44) = 25.86, *p* < 0.0001) and feedback (*F*(1, 44) = 67.25, *p* < 0.0001) on criterion and a significant cross-over interaction (*F*(1, 44) = 23.71, *p* < 0.0001). Pairwise comparisons show a strong LPE effect in the form of a conservative criterion shift when feedback is given (*t*(11) = 6.67, *p* < 0.0001). Without feedback, no significant change in criterion is observed on average when going from high to low prevalence (50% prevalence: 0.89, 20% prevalence: 0.91; *t*(11) = 0.20, *p* = 0.84). After performing the feedback condition first, observers carried the conservative criterion to the subsequent no-feedback high prevalence block (criterion significantly deviates from 0: *t*(11) = 13.17, *p* < 0.0001). The conservative criterion is sustained throughout the no-feedback high prevalence block. There is no significant change in criterion between the first 60 trials (0.83) and the last 60 trials (0.91) in the no-feedback high prevalence block (t(11) = −0.66, *p* = 0.53), suggesting that observers did not adjust their criterion when the blast cell prevalence increased in the absence of feedback. There is no effect of prevalence or feedback on d’, nor a cross-over interaction (all *p* > 0.28). The d’ values of the novices are relatively low, but they are above chance, confirming that the novices can distinguish blast and non-blast cells at above chance levels.

The main effects are the same when the feedback condition comes second (*n* = 13). A two-way ANOVA with prevalence and feedback as factors shows significant effects of both prevalence (*F*(1, 48) = 7.36, *p* = 0.009) and feedback (*F*(1, 48) = 8.03, *p* = 0.007) on criterion and a cross-over interaction (*F*(1, 48) = 20.29, *p* < 0.0001). Feedback produces an LPE effect (*t*(12) = 8.80, *p* < 0.0001). Without feedback, the criterion becomes slightly more liberal on average (50%: 0.04, 20%: −0.09), but this is not statistically significant (*t*(12) = 1.03, *p* = 0.33). When observers do not experience the feedback condition first, the starting criterion in the no-feedback 50% prevalence condition does not deviate significantly from 0 (*t*(12) = 0.42, *p* = 0.68). Again, there are no effects on d’.

Overall, Experiment 1 with novice observers replicates previous findings: Low target prevalence affects decision criterion, and this effect is modulated by the presence and absence of feedback. As shown in Fig. 2, an initial exposure to feedback seems to set the criterion for the subsequent no feedback condition.

## Experiment 2: blast cell detection with medical professionals

In Experiment 2, we recruited medical professionals to participate in the blood cell identification experiment as in Experiment 1. The key question here is whether prevalence and feedback affect novices and experts similarly. The interaction between low prevalence and expertise has been studied in other contexts (Evans et al., 2013; Wolfe et al., 2013), although few, if any, studies have directly manipulated the presence and absence of feedback when studying expert populations. Experts certainly show low prevalence effects, but it is not known whether feedback modulates those effects. The extensive experience and knowledge may enable experts to respond to feedback and mistakes differently. To anticipate the results, Experiment 2 provides evidence that experts and novices show similar effects of low prevalence and feedback.

### Methods

#### Preregistration

Experiment 2 was preregistered on the Open Science Framework (https://osf.io/u2tnp/) where raw data files are publicly available.

#### Observers

Experts were recruited through a mass email posted to a list, purchased from the American Society for Clinical Pathology. This produced a convenience sample of 24 observers with varying degrees of expertise (see Table 2). It is, indeed, ‘convenient’ that the yield of 24 experts was in-line with our power calculations.

**Table 2:** Observers divided by their role and training

Role and training	Number of observers
Medical technologist	16
Pathologist (Trained in Hematopathology)	2
Pathologist (Not Trained in Hematopathology)	2
Medical laboratory scientist	2
Educator	1
Choose not to report	1

Observers were asked to report their area of responsibility (Table 3).

**Table 3:** Observers divided by their area of responsibility

Area of responsibility	Number of observers
Hematology/Coagulation	11
Clinical Pathology	4
Generalist	3
Transfusion Medicine/Blood Banks	2
Hematopathology, Surgical Pathology and Genetics	1
Teaching	1
Choose not to report	2

They also reported the approximate number of blood smears they reviewed annually (Table 4). Three observers who reported reviewing zero blood smears per year are removed from further analysis. Removing the 3 observers does not change the results in any substantial manner (See Supplementary Materials).

**Table 4:** Reported number of blood smears reviewed by observers annually

Number of blood smear review	Number of observers
> 1000 per year	9
500–1000 per year	3
0–500 per year	9
0 per year	3

For the remaining 21 observers, there were 6 male and 15 female with an average age of 52 ± 16 (6 observers did not report their age). Detailed demographical information can be found on the experiment OSF page. Observers attested to 20/25 vision with correction and color vision. All participants were entered into a contest and the participant with the highest performance was awarded a $150 gift card prize. All data were de-identified. Participants gave informed consent. Procedures were approved by the Institutional Review Board at Brigham and Women’s Hospital (IRB #2007P000646). All observers were naïve to the purpose of the task.

#### Stimuli and methods

The experiment design was identical to Experiment 1 except that the two training blocks were omitted and observers directly entered the main experiment. Observers participated with the URL we provided and were not monitored by experimenters. The experiment took approximately 40 min for the medical specialists to finish.

### Results

We first provide results for the experts only and then compare experts and novices.

#### Experts only

Trials with reaction times shorter than 200 ms and longer than 4000 ms (< 1% of all trials) were removed from further analysis. The average accuracy of the 21 observers in Experiment 2 was 79.7% (Feedback: 80.1%, No-Feedback: 79.3%). The accuracy prior to removing the three observers who reported reviewing 0 blood smears annually was 79.3%.

The lower panels of Fig. 2 show criterion and d’ separately for prevalence X feedback condition X feedback order. The basic pattern of results is similar to that seen with novices. When the feedback condition comes first (*n* = 12), a two-way ANOVA with prevalence and feedback as factors shows a significant effect of feedback (*F*(1, 44) = 26.12, *p* < 0.0001) on criterion. There is no significant effect of prevalence (*F*(1, 44) = 1.78, *p* = 0.19) and a moderate cross-over interaction (*F*(1, 44) = 3.49, *p* = 0.068). Pairwise comparisons between the high and low prevalence block shows a conservative criterion shift when feedback is given (*t*(11) = 3.57, *p* = 0.0044) and no shift without feedback (50% prevalence: 0.55, 20% prevalence: 0.51; *t*(11) = 0.46, *p* = 0.65). Again, after performing the feedback condition first, the criterion level at the no-feedback 50% prevalence condition is shifted significantly in the conservative direction (compare with c = 0: *t*(11) = 7.36, *p* < 0.0001). Criterion between the first 60 trials and the last 60 trials in the no-feedback high prevalence block are not significantly different (*t*(11) = −1.27, *p* = 0.23), suggesting that expert observers did not adjust their criterion in the new prevalence environment in the absence of feedback. There is no effect of prevalence or feedback on d’, nor a cross-over interaction (all *p* > 0.71).

When the feedback condition comes second (*n* = 9), a two-way ANOVA with prevalence and feedback as factors shows no significant effect of feedback (*F*(1, 32) = 1.39, *p* = 0.25), prevalence (*F*(1, 32) = 0.59, *p* = 0.45), nor a cross-over interaction (*F*(1, 32) = 1.59, *p* = 0.22). Pairwise comparisons between the high and low prevalence block indicates a conservative criterion shift when feedback is given (*t*(8) = 3.23, *p* = 0.012) and no shift without feedback (50% prevalence: 0.37, 20% prevalence: 0.31; *t*(8) = 0.57, *p* = 0.58). Interestingly, the medical specialists show a conservative criterion at the start of the experiment (no feedback high prevalence condition), without having experienced an initial feedback block which has been demonstrated to bias response criterion for the subsequent block. Their criterion in the no feedback high prevalence condition significantly deviates from 0 (*t*(8) = 2.60, *p* = 0.032). This is different from novices in Experiment 1 and previous studies using simple stimuli (Lyu et al., 2021). This starting conservative criterion may reflect a low prevalence ‘prior’ that the medical specialists may have brought to the experiment based on their experience of a low prevalence environment. However, we do not know the base rate of blast images in the clinical context. There is no effect of prevalence or feedback on d’, nor a cross-over interaction (all *p* > 0.20). When observers experience the no feedback block first, in the absence of feedback, reducing prevalence from 50 to 20% improves d’ (*p* = 0.013). It is not clear why. One explanation is that 20% prevalence is more similar to the experts’ internal setting and expectation than 50%. At 50% prevalence, without knowing that the base rate is high, experts may be unwilling to report positive findings as they may raise the false positive rate, and as a result, their d’ drops.

Is there any relationship between the magnitude of the criterion shift and the number of blood smears reviewed by observers? It is possible that experts who have reviewed a lot of blood smears are more confident in their decisions and less responsive to negative feedback, and thus less likely to adjust their decision criterion when provided with feedback. To investigate the effect of expertise on criterion shift, we compared the criterion shift between experts who reported reviewing < 500 blood smears per year (Low Group, *n* = 9) and those who reported reviewing > 1000 blood smears per year (High Group, *n* = 9). We found that both groups showed a significant shift in criterion with feedback (paired t-test; Low Group: t(8) = 3.18, *p* = 0.013; High Group: t(8) = 3.36, *p* = 0.010) and little shift in criterion without feedback (Low Group: t(8) = −1.27,* p* = 0.24; High Group: t(8) = −0.93, *p* = 0.38). Therefore, our result suggests that having more experience reviewing blood smears does not significantly reduce criterion shift with feedback in this setting.

#### Comparison of novices and experts

The medical specialists demonstrated significantly higher performance accuracy compared to the novices (*t*(43.88) = 7.21, *p* < 0.0001). Across all 8 block types (2 feedback orders × 2 feedback types × 2 prevalence levels), experts consistently showed higher d’ values than the novices. Two-sample *t*-tests revealed that these differences were statistically significant across all blocks (*p* < 0.0001), except for the 'feedback-second, no-feedback, high-prevalence' block, where the difference was marginally significant (*t*(20) = −1.90, *p* = 0.072).

**Fig. 3:**
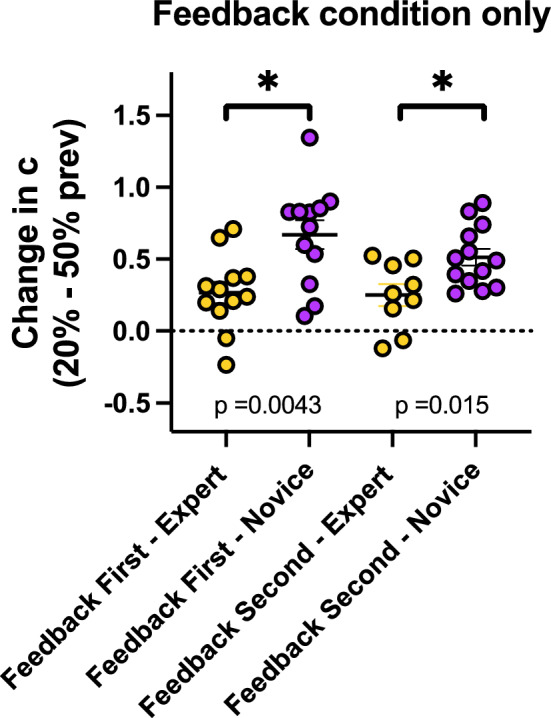
Experts show a smaller criterion shift than novices in the feedback condition. Figure plots the change in criterion from 50% to 20% prevalence block in the feedback condition, separately for experts and novices, and observers who received feedback first and second. *P*-values show results for an unpaired t-test with Welch's correction, testing against the null hypothesis that there is no difference in change in criterion. Error bars show ± 1 SEM

Next, we examined whether low prevalence shifts expert and novice observers’ criterion with equal magnitude in the feedback condition. An unpaired t-test with Welch’s correction (Fig. 3) shows that criterion change from high to low prevalence in the feedback condition is less dramatic in experts than in novices, when the feedback condition comes first (*t*(20.40) = 3.21, *p* = 0.0043) and second (*t*(16.19) = 2.71, *p* = 0.015). Thus, although experts show an LPE, its magnitude may be dampened by the experience they bring to the task. Experts may have stabilizing priors that novices would lack.

To further assess the effect of expertise and its interaction with the effects of prevalence and feedback, we fit a linear model to the criterion value with three predictors, expertise, feedback, and prevalence, and their interaction plus a fixed intercept. Table 5 shows the results of the regression analysis. There was no effect of expertise, feedback, or prevalence. However, the interaction between expertise and feedback was significant, suggesting that the effect of feedback on criterion depends on the level of expertise. The interaction between feedback and prevalence was also significant, confirming the basic finding that feedback modulates the effects of change in prevalence. The effects of the three predictors—expertise, feedback, and prevalence—on d’ were also assessed with a linear model. Only expertise was a significant predictor of d’, confirming that medical professionals out performed the novice in this cancer cell detection task. None of the other predictors or interaction terms were significant.

**Table 5:** Results of linear models examining the effects of expertise, feedback, and prevalence on two dependent variables: criterion (top) and d′ (bottom). Each table presents the model estimates, 95% confidence intervals (CI), and p-values for main effects and interactions. Significant predictors are highlighted in bold. The adjusted R^2^ values indicate model fit

*Predictors*	*Estimates*	*CI*	*p*
*Criterion*			
(Intercept)	0.39	0.24–0.54	**< 0.001**
Expertise	0.04	−0.19–0.26	0.745
Feedback	0.16	−0.05–0.38	0.130
Prevalence	0.06	−0.15–0.27	0.581
Expertise:Feedback	−0.32	−0.63– 0	**0.047**
Expertise:Prevalence	−0.01	−0.32–0.30	0.953
Feedback:Prevalence	−0.65	−0.95– −0.35	** < 0.001**
Expertise:Feedback:Prevalence	0.34	−0.11–0.78	0.135
Observations	184		
R^2^/R^2^ ADJUSTED	0.24/0.21		
*D’*			
(Intercept)	0.73	0.56–0.90	** < 0.001**
Expertise	0.88	0.63–1.14	** < 0.001**
Feedback	−0.14	−0.39–0.10	0.257
Prevalence	0.06	−0.19–0.30	0.660
Expertise:Feedback	0.14	−0.22–0.50	0.454
Expertise:Prevalence	−0.21	−0.57–0.15	0.251
Feedback:Prevalence	0.01	−0.33–0.36	0.945
Expertise:Feedback:Prevalence	0.06	−0.46–0.57	0.830
Observations	184		
R^2^/R^2^ adjusted	0.51/0.49		

## Discussion

The two versions of the blood cell classification experiment with novice and expert observers bring together prior findings concerning the effect of prevalence, availability of feedback, and expertise on decisions. Prior knowledge and experience of the low target base rate is another factor that can influence decision-making in low prevalence conditions (Trueblood et al., 2021), but it is not the focus of the present study. Observers were not informed of the target base rate in our task. We found that with trial-by-trial accuracy feedback, low prevalence produces a classic LPE. Observers become more conservative in their decisions. Without feedback, the LPE was absent in this experiment. Note that we define LPE as an increase in observers’ decision criterion (more conservative) when target prevalence decreases. We did not observe significant *changes* in the criterion in either novice or expert groups in the absence of feedback. If anything, the criterion shift is in the opposite direction, although the liberal PICC criterion shift was not statistically significant in this experiment. The lack of LPE without feedback in this experiment does not contradict the fact that clinicians can adopt a conservative criterion in clinical settings where feedback is rare or not possible, based on their knowledge or experience about the low disease base rate. Nonetheless, our findings suggest that clinicians may be less likely to adjust their criterion in response to a change in target prevalence if immediate feedback is not available. These results also show that the effects of low target prevalence and feedback are not restricted to simple laboratory stimuli that vary along one feature continuum (Lyu et al., 2021) but generalize to more complex real-world stimuli.

Unsurprisingly, medical professionals perform better than novices on this task as shown in their higher d’ values in Fig. 2. However, experts and novices are both affected by low prevalence. The effect is weaker in experts (Fig. 3), suggesting expertise may modulate the size of the LPE. This is congruent with other low prevalence effects studies with expert populations (Evans et al., 2011, 2013; Growns & Kukucka, 2021; Wolfe et al., 2013). For novices, experiencing one feedback block shifts the starting criterion in the conservative direction in the subsequent no feedback block. This is not seen when the no feedback block is performed before the feedback block. This experience dependent effect was also reported by Lyu et al. (2021) and systematically studied by Wolfe (2022) where it was found that a block of low prevalence trials with feedback produced a more conservative criterion on a subsequent block of trials on average, while a block of higher prevalence trials with feedback produced a more liberal criterion on a subsequent block of trials. Blocks of no feedback trials did not have reliable effects on the next block of trials.

In the results reported here, experts demonstrate a conservative criterion at the start of the experiment if they do not receive feedback. Experts’ conservative initial criterion may reflect their education or experience working in a low prevalence clinical environment. Prevalence in clinical settings can vary quite widely. For instance, in many large hospitals, the initial hematopathology screening would be done by an automated image analyzer. A technologist would see a relatively enriched set of images and, based on their processing, the MD pathologist would see a set of images with an even higher baseline level of pathology. At the same time, all involved might know that the base rate in the initial population is lower than what they are seeing. Similarly, in radiology, medical images may be triaged based on the urgency level. The prevalence of pathology likely differs between images in urgent and non-urgent image pools.

Even though the criterion shifts in novices and experts are in the same direction, the cognitive mechanisms that underlie this shift could differ between groups. For example, Trueblood et al. (2021) found similar criterion shifts in experts and novices when prevalence was manipulated. However, further analyses using the decision diffusion model (DDM) revealed a response bias (shift in the DDM starting point) in novices and a stimulus evaluation bias (shift in the DDM drift rate) in experts. Trueblood et al. propose that the response bias in novices predominantely comes from observers’ strategy to choose blast or non-blast responses based on how often they think the blast cell occurs. Whereas in experts, a more pronounced stimulus evaluation bias suggests that prevalence alters how observers evaluate each image. Lyu et al. (2021) suggest that the criterion shift with feedback may come from observers’ tendency to balance false positive and false negative errors. At 50% prevalence, observers respond target present and target absent with approximately equal probability. When target prevalence decreases, feedback informs observers that the errors are predominately false positive errors. To reduce the false positive error, observers say target present less often and become more conservative. This explanation is similar to the response bias account in Trueblood et al. where observers develop a response strategy based on prevalence.

Our task with different target prevalence rates is relevant to real-life situations where target prevalence varies across settings. For example, disease prevalence might change if a radiologist moves from teaching to clinical settings or goes from a referral hospital to a primary care clinic. The prevalence of disease in medical images might change during a disease outbreak and would vary across seasons for some diseases. Disease prevalence is also known to vary based on patient demographics and associated risk factors. We do not always know the expected prevalence (e.g., I may not know the prevalence of crime in a new city when I judge if a person is suspicious). Or the expected prevalence may not match the actual prevalence. Nonetheless, prior expectations can have an impact on diagnostic efficacy (Littlefair et al., 2016). Observers need to constantly adapt to the actual prevalence, using external feedback when it is available or through their own response rate when direct feedback is not available. In the blast cell detection task, we found that both novices and experts were only able to adjust their criteria when feedback was present. Providing feedback did not improve d’ in the main experiment, suggesting that neither group showed additional learning of the stimuli or the reference standard.

The study has several limitations. First, the cell difficulty continuum used in the current study was based on the average difficulty ratings of three pathologists rather than an objective measure as is possible with, for example, a blue-purple color continuum. The image that one pathologist finds difficult or ambiguous may not be considered as difficult/ambiguous by another viewer, and vice versa. This imperfect classification standard could add noise to the data. As mentioned in the introduction, the presence or absence of a clear reference standard in stimuli may influence the effects of low prevalence, and particularly PICC. Thus, the results we report here on the effects of expertise with cell images, especially in the no feedback condition, may not extend to tasks using stimuli with either no reference standard, or a clearly defined reference standard. Second, our study uses 748 images of cells. Limited by the number of available images, a fraction of the images were presented more than once to the same observers to ensure enough trials. Additionally, some images were used in both the training and the testing blocks. If anything, the reuse of a training image for testing would only reduce any effect of prevalence and criterion shift. Removing the repeated images did not affect the main findings (see Supplementary Materials). Thirdly, we did not take the variability of the cell images into account when powering our study, so the result may not generalize to readers viewing a different set of images. It is also worth noting that the majority of the medical experts in this study were medical technologists, and only two were MD pathologists. It is not clear that this is a limitation, as most of this type of cell classification task would be performed by medical technologists who are likely to be as expert a group as any on this task. It might be interesting for future studies to compare performance among expert populations as the medical specialist populations may be working in different prevalence contexts. It may also be worth testing a larger sample size of experts with different levels of experience to examine whether more experience makes one less susceptible to low prevalence effects. The use of MD experts may be of particular interest when studying more complex decisions. Imagine if the MD’s decision involved evaluating multiple sources of information, such as patient history and medical images. It would be interesting to know how such decisions are influenced by prevalence and/or feedback.

In conclusion, this study found that the effects of low target prevalence and feedback occur in a real-world blast cell identification task, for both novices and experts. In the presence of feedback, observers adopted conservative decision criteria and showed a Low Prevalence Effect. Without feedback, neither experts nor novices made significant adjustments to their decision criterion when target prevalence was reduced. This is consistent with other findings of prevalence effects in expert populations and suggests that although expertise may modulate effects of low prevalence, expertise does not eliminate these effects.

## Supplementary Information

Supplementary material 1.

## Data Availability

The raw datasets generated and analyzed during the current study are available in the Open Science Framework repository. Link to Experiment 1: https://osf.io/k2ngv/; Experiment 2: https://osf.io/u2tnp/.
