# Supplementary material for: Effects of prevalence and feedback in the identification of blast cells in peripheral blood: expert and novice observers
Source: Cogn Res Princ Implic. 2025 Jun 15;10:30. doi: 10.1186/s41235-025-00632-7 (PMC12167727; doi:10.1186/s41235-025-00632-7)
Supplement: Supplementary file 1 — Supplementary material 1. [file 41235_2025_632_MOESM1_ESM.docx]

Supplementary Materials for

**Effects of prevalence and feedback in the identification of blast cells**

**in peripheral blood: expert and novice observers**

Wanyi Lyu^a,*^, Jennifer S. Trueblood^b^, Jeremy M. Wolfe^c,d^

1. Department of Biology, Centre for Vision Research, York University, Canada
2. Department of Psychological and Brain Sciences and Cognitive Science Program, Indiana University, Bloomington, USA
3. Visual Attention Lab, Department of Surgery, Brigham & Women's Hospital, Boston, MA
4. Departments of Ophthalmology & Radiology, Harvard Medical School, USA
5. **Main analysis limited to repeated trials**

To check if repeated images influenced the effects of prevalence and feedback, we examined the results when analyses were limited to only the repeated trials. Because most repeats occur in the second part of the experiment, to ensure sufficient trials for both high and low prevalence conditions for comparison, we narrowed the analysis to the second half of the experiment. That is, we analyzed the no-feedback condition from the Feedback First group and the feedback condition from the Feedback Second group.

Experiment 1 – Novice observers:

With feedback (Feedback Second Group, n = 13), pairwise comparisons show a strong LPE effect in the form of a conservative criterion shift (50% prevalence: -.13, 20% prevalence: .42; t(12) = 5.77, p < .0001). Without feedback (Feedback First Group, n = 12), no significant change in criterion is observed on average when going from high to low prevalence (50% prevalence: .85, 20% prevalence: .90; t(11) = .54, p = .60). There is no effect of change in prevalence on d’, with or without feedback (all p > .23). Thus, novice results limited to the repeated images are consistent with the main finding reported in the MS. Repeating image does not alter the main findings.

Experiment 2 – Expert observers:

With feedback (Feedback Second Group, n = 9), pairwise comparisons show a significant criterion shift as prevalence decreases (50% prevalence: .072, 20% prevalence: .35; t(8) = 2.53, p < .05). Without feedback (Feedback First Group, n = 12), no significant change in criterion is observed (50% prevalence: .69, 20% prevalence: .59; t(11) = 1.30, p = .22). There is no effect of change in prevalence on d’, with or without feedback (all p > .37). Thus, expert results limited to the repeated images are also consistent with the main finding reported in the MS.

1. **Experiment 2 – analysis including three excluded pathology experts who reported reviewing 0 blood smears annually**

Including the data from pathologists who reported reviewing 0 blood smears annually did not alter the main results in the MS.

When the feedback condition comes first (n = 14), a 2-way ANOVA with prevalence and feedback as factors shows a significant effect of feedback (*F*(1, 52) = 25.92, *p* < .0001) on criterion. There is no significant effect of prevalence (*F*(1, 52) = 1.48, *p* = .23) and a moderate cross-over interaction (*F*(1, 52) = 2.93, *p* = .093). Pairwise comparisons between the high and low prevalence block show a conservative criterion shift when feedback is given (*t*(13) = 3.32, *p* < .01) and no shift without feedback (50% prevalence: 0.52, 20% prevalence: 0.48; *t*(13) = 0.48, *p* = .64). There is no effect of prevalence or feedback on d’, nor a cross-over interaction (all *p* > .47).

When the feedback condition comes second (n = 10), a 2-way ANOVA with prevalence and feedback as factors shows no significant effect of feedback (*F*(1, 36) = 2.58, *p* = .12), prevalence (*F*(1, 36) = 0.85, *p* = .36), nor a cross-over interaction (*F*(1, 36) = 1.23, *p* = .28). Pairwise comparisons between the high and low prevalence block indicate a conservative criterion shift when feedback is given (*t*(9) = 3.32, *p* < .01) and no shift without feedback (50% prevalence: 0.40, 20% prevalence: 0.38; *t*(9) = 0.23, *p* = .83). There is no effect of prevalence or feedback on d’, nor a cross-over interaction (all *p* > .19).
